# The Interplay between Scientific Overlap and Cooperation and the Resulting Gain in Co-Authorship Interactions

**DOI:** 10.1371/journal.pone.0137856

**Published:** 2015-09-15

**Authors:** Itay Mayrose, Shiri Freilich

**Affiliations:** 1 Department of Molecular Biology and Ecology of Plants, Tel Aviv University, Tel Aviv, 69978, Israel; 2 Newe-Ya'ar Research Center, Institute of Plant Sciences, Agricultural Research Organization, PO Box 1021, Ramat Yishay, 30095, Israel; VU University Amsterdam, NETHERLANDS

## Abstract

Considering the importance of scientific interactions, understanding the principles that govern fruitful scientific research is crucial to policy makers and scientists alike. The outcome of an interaction is to a large extent dependent on the balancing of contradicting motivations accompanying the establishment of collaborations. Here, we assembled a dataset of nearly 20,000 publications authored by researchers affiliated with ten top universities. Based on this data collection, we estimated the extent of different interaction types between pairwise combinations of researchers. We explored the interplay between the overlap in scientific interests and the tendency to collaborate, and associated these estimates with measures of scientific quality and social accessibility aiming at studying the typical resulting gain of different interaction patterns. Our results show that scientists tend to collaborate more often with colleagues with whom they share moderate to high levels of mutual interests and knowledge while cooperative tendency declines at higher levels of research-interest overlap, suggesting fierce competition, and at the lower levels, suggesting communication gaps. Whereas the relative number of alliances dramatically differs across a gradient of research overlap, the scientific impact of the resulting articles remains similar. When considering social accessibility, we find that though collaborations between remote researchers are relatively rare, their quality is significantly higher than studies produced by close-circle scientists. Since current collaboration patterns do not necessarily overlap with gaining optimal scientific quality, these findings should encourage scientists to reconsider current collaboration strategies.

## Introduction

Cooperative behavior is considered essential for the development of culture and society. In contemporary science, raising financial support and conducting high-profile research rely heavily on the construction of a versatile and often multi-disciplinary team of researchers [1–3]. The flipside of cooperation–conflict or competition–appears to be not less common component of social and scientific life. Scientific progression is to a large extent dependent on the interplay between these two behavior types. Competition has undoubtedly been a driving force to key breakthroughs. One of numerous examples is the publication of *On the Origin of Species* that was expedited following Darwin’s introduction to Wallace’s work [4]. Nonetheless, successful team work is also a major contributor to scientific productivity allowing the sharing of resources and work load, and encourages the flow of skills and ideas [5–10]. The effectiveness of a collaborative research group is dependent upon multiple factors including its size, the intellectual interactions among its members, and the diversity among team members [8,11]. Notably, collaborative interactions are not always beneficial and, for example, can lead to a diffusion of responsibilities or to undermining the contribution of junior partners [3]. Since successful collaborative interactions are expected to raise the creative potential and the productive capacity of group members, setting the conditions that produce optimal cooperation structure will allow to optimize the effectiveness of research groups [8,11–13].

Systematic approaches for the screening of large scale scientific repositories and automatic data retrieval have been previously applied for the construction of collaboration networks based on co-authorships of peer-reviewed articles. This allowed linking topological properties such as centrality and modularity with scientific productivity and quality [13–21]. Here, taking a similar approach for defining collaborative interactions, we tackled basic conflicts in the establishment of scientific collaborations by associating independent estimates for the extent and quality of different interaction types between researchers. Four interaction parameters were collected for pairs of scientists belonging to the biological sciences field from ten of the top academic institutions worldwide: (i) the relative scope of their collaborative work, (ii) the scientific impact of the collaboration, (iii) the extent of overlap in their research interests, and (iv) their professional social circles (institutional affiliations). Making use of these interaction estimates, we explored the tendency to collaborate and the typical gain of the collaboration considering the scientific and administrative circles of the collaborators aiming at addressing two key questions: (i) with whom do scientists tend to collaborate? and (ii) with whom should they collaborate?

## Materials and Methods

### Data assembly

#### Compilation of affiliation list and publication record retrieval

A list of the 10 top universities ranked under the biological sciences category was retrieved from THE-QS World University Ranking [22]. These include the Massachusetts Institute of Technology (MIT), California Institute of Technology (Caltech), the universities of Harvard, Stanford, Cambridge, Oxford, Yale, and the University of California at Berkeley, Los Angeles (UCLA) and San Diego (UCSD). For each university, departments belonging to the biological sciences field were identified using manual web searches. Focusing on the top 10 universities encompassing 40 departments allowed rigorous data validation, such as correctly resolving ambiguous names–a problem for which no standard solution exists [23–26], yet encompassing a substantial amount of data (ca. 1000 researchers and 20,000 research articles).

For each department, search queries conducted through Thomson Reuters (formerly *ISI*) *Web of Knowledge* were used to retrieve scientific articles published in the time period 2000–2012 by authors affiliated with the queried department. Search results were manually surveyed to verify their reliability. Publication records were parsed to obtain for each publication its doi number and the names and affiliations of all co-authors. This allowed the identification of authors affiliated with the queried department and the construction of researcher-specific list of publications (termed PUB_ALL), as well as a list of publications in which the researcher appeared last (PUB_LAST). Due to various author name ambiguities (differences in naming conventions, data entry errors, or distinct individuals possessing identical names), the task of obtaining the genuine and complete list of publications for all authors is a challenging one [26]. To this end, the article lists of authors from the same department with similar names were combined if their names did not contradict (e.g., combining Ullman, David with Ullman, D. M. and with Ullman, D. but not with Ullman, Daniel M. or with Ullamn, D. C.). Further, we searched for scholars affiliated with multiple departments and united their articles list. To this end, authors of different departments but with identical last name and first name initials were identified and identities were manually verified using web searches. This allowed us to identify researchers affiliated with multiple departments within a single institution, as well as six researchers that were affiliated with more than a single institution during the time period 2000–2012 (these include either change of institution or multiple affiliation). Finally, the full list of authors was manually scanned to verify that all authors with matching last name and first name initials are indeed distinct authors (e.g., Blackman T. L. from Yale University is distinct from Blackman Tim from Stanford University) and to identify authors with naming incongruences. Three such cases were identified (Wagner, Guenter P. and Wagner, Gunter P.; Deng, Xing-Wang and Deng, Xing Wang; Levine, Michael S. and Levine, Mike) and their respective publication lists were united.

To ensure that the list of researchers generally includes principle investigators, only researchers with at least five publications in PUB_LAST were considered [e.g., similar to 19]. This procedure resulted in a total of 12,838 and 18,801 publications belonging to PUB_LAST and PUB_ALL, respectively, and encompassing a total of 937 researchers affiliated with 40 departments from the 10 universities (S1 Table). These two author-specific lists were then used to construct author versus author matrices of collaboration and research overlap, based on the lists PUB_ALL and PUB_LAST, respectively (see below).

### Calculating pairwise interaction scores

#### Calculating a pairwise collaboration score (CLS)

For all possible pairwise combinations of authors we have calculated an a-symmetrical collaboration score, computed as follows:

$${CLS}_{ij}=\frac{\sum_{n\in P_{i}}\;1\left(if\;n\in \;P_{j}\right)}{N_{i}}$$

where *P*_*x*_ is the full publication record (PUB_ALL) of author *x* and *N*_*x*_ is the number of publications in that list. That is, the collaboration score of author *i* with author *j* is the fraction of their joint publications out of all publications of author *i*.

#### Calculating a pairwise research-overlap score (ROS)

The scientific interest of each researcher was determined based on the distribution of keywords associated with its publications. Publication-specific keywords were based on the Medical Subject Headings (MeSH) terms associated with the publication. MeSH terms were used since they provide a controlled vocabulary that can be matched across multiple publications (unlike keywords that are usually personally entered as free text). For each researcher, scientific interests were determined based on the list of publications in which the researcher is the last author (PUB_LAST). In the biological sciences field, the principle investigator (PI; here referred to as the author who initiates, leads, and funds the study) is conventionally placed as the last author, and hence publications in PUB_LAST are most indicative of the PI research interest rather than the full list of its publications. For each publication in PUB_LAST, we have used the doi number to automatically retrieve the corresponding PubMed record and its associated MeSH terms. For each author, a list of MeSH terms was constructed filtering those whose overall appearance in PUB_LAST is lower than a certain threshold. For the results reported in the main text a cutoff of 4 was used; other cutoffs yielded similar observations (see S1 Fig). The weighted list of MeSH terms thus represents the key scientific niche of the corresponding PI; high overlap between two MeSH term distributions suggests similar research interests between the corresponding PIs while little overlap suggests absence of mutual research interests.

For all possible pairwise combinations of authors we have calculated an a-symmetrical research overlap score (ROS), computed as follows:

$${ROS}_{ij}\;=\;\sum_{n=1}^{N}\frac{\text{min}\left\{M_{ni},M_{nj}\right\}}{M_{ni}}$$

where *N* is the set of all MeSH terms and *M*_*nx*_ is the number of times the MeSH term *n* appeared in papers associated with author *x*. That is, the research overlap score of author *i* with author *j* is the fraction of their joint MeSH terms (out of the total number of MeSH terms associated with author *i*), taking into account the number of times each MeSH term appeared. Alternative calculations of the research overlap score, described in S2 Fig, lead to similar observations.

#### Assessing pairwise impact score (IS)

The quality of publications was estimated using two different journal metrics: the Source Normalized Impact per Paper (SNIP) [27,28], and the SCImago Journal Rank (SJR) [29]. Both scores were designed to correct for differences in citation practices and potentials between scientific fields (for example by weighting the relationships between the citing and cited journals) and hence found to be appropriate for studies involving cross-disciplines analyses, such as the one conducted here, compared to the widely used Thomson Reuters Impact Factor measure [30]. We note that the impact score of the journal provides a direct estimate to the prestige of the journal, which is another dimension to evaluate the collaboration success. We also note that citation count of the articles *per se* can also be used to determine publication quality. However, such a measure does not account for different citation practices (and potentials) across fields as well as introduces publication date biases (favoring older articles) and hence is unsuitable for this specific data set.

The pairwise impact score (IS) of researchers *i* and *j* was calculated as the mean impact value (SNIP/SJR) of their joint publications. Results obtained using the two measures were qualitatively similar, thus, our results are presented using the SJR index only.

### Statistical analyses

All statistical analyses were performed using the R platform. The distributions of CLS and IS across different ROS bins were compared in a Wilcoxon rank sum test using the wilcox.test function in the R platform. Values were adjusted for multiple comparisons using the false discovery rate (FDR) correction [31]. CLS values: CLS values were compared between all pairwise bin combinations using a one-sided Wilcoxon test. IS values: IS values within each bin were compared to the overall distribution of IS values in the data. In addition, IS values were compared between all pairwise bin combinations using one-sided Wilcoxon test.

## Results and Discussion

Overall, a list of nearly 1000 researchers affiliated with the biological sciences departments of 10 highly-ranked institutions was compiled. All institutions are located in the US (8) and UK (2) and the surveyed departments spans a range of biological disciplines including molecular biology, biochemistry, ecology, plant science, zoology, and systems biology (see Methods, S1 Table). For each researcher, a weighted list of MeSH terms was constructed, representing the key scientific niches of its professional activity (Methods).

**Fig 1:**
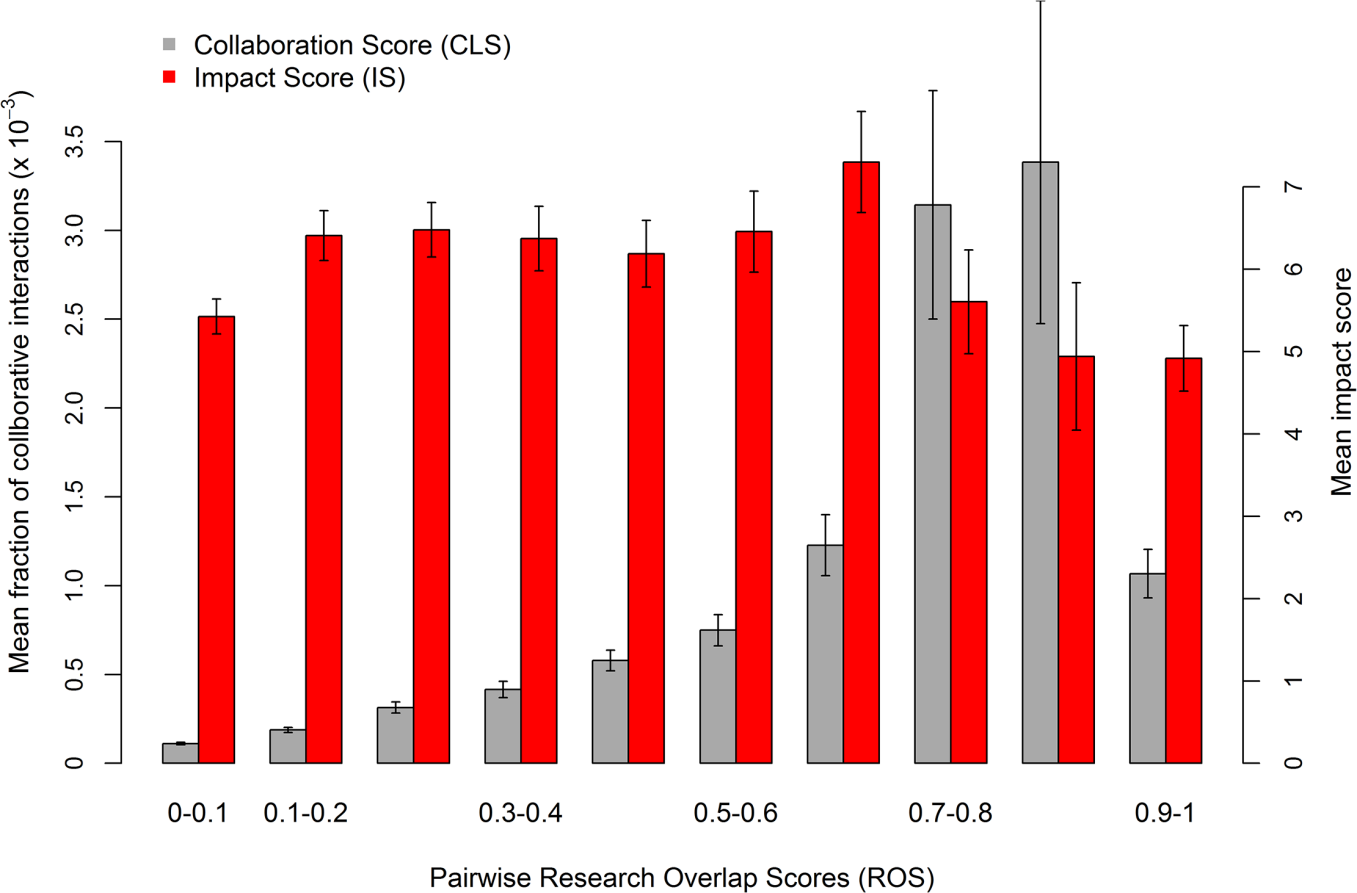
The relative abundance and scientific impact of collaborative interactions versus the level of research overlap. Distribution of pairwise scores for estimating the fraction of collaborative interactions (CLS) and their scientific impact scores (IS) across different level of pairwise research overlap (ROS). Pairwise scores were computed for 368511 non redundant pairs of researchers (Methods). The number of researchers pairs that fall in each bin (low to high ROS): 400664, 129032, 93748, 46361, 25874, 26797, 8825, 2970, 2240, 26491. Mean IS score were considered only for collaborating pairs (hence reflecting collaboration quality and not tendency). The number of collaborating pairs in each bin (low to high ROS): 558, 360, 312, 208, 165, 143, 85, 50, 31, 138. Bars represent standard error.

Research interactions formed between pairwise combinations of these scientists were described by two quantitative estimates: the tendency of collaborative interactions as reflected by the number of co-authored publications (Collaboration Score, CLS), and the overlap in scientific interests estimated according to the overlap in key MeSH terms (Research Overlap Score, ROS; see Methods). Given these two independent scores, we then examined the fraction of collaborative interactions across different levels of overlap in scientific interests. The results, shown in Fig 1, demonstrate that the highest fraction of collaborative interactions is obtained at moderate to high levels of overlap in scientific interests, while at the highest degree of interest overlap, as well as in lower ones, the tendency to collaborate declines. Collaborative interactions are highest at bins 8–9, significantly more frequent than at bins of lower (bins 1–6, left; *p*-value < 0.0001 for all pairwise comparisons) and higher (bin 10, right; *p*-value < 1×10^−6^) ROS bins (Methods). That is, scientists tend to collaborate more often with colleagues with whom they share a moderate level of mutual interests and knowledge. The cooperative tendency declines at the highest level of resource overlap (with respect to the scientific niche the pair members occupy) suggesting low synergistic potential, and at the lower levels suggesting communication gaps.

To examine whether this pattern is consistent across professionally distinct subcategories, we compared the CLS-ROS interplay at groups of scientists divided according to their methodological discipline (genetics, computational, molecular) and according to the model organism employed (human, plant, bacteria). While the limited category-specific pairs in each bin preclude a statistically robust interpretation of the data, similar trends regarding the CLS-ROS interplay were qualitatively observed: at all cases, a decline is observed at ROS values both higher and lower than the ROS bin with a maximal collaboration tendency (S3 Fig).

While the distribution pattern of collaboration scores delineates with whom researchers tend to collaborate, it does not test the scientific value of the resulting collaborations. Exploring the scientific impact of collaborative research (as measured by two alternative impact scores, IS, of the resulting articles; Methods) as a function of scientific interests overlap, we observed that the decline in the fraction of collaborative interactions across the ROS gradient is not followed by a corresponding decline in the quality of collaboration, where none of the bins were found to significantly differ in their IS values compared to the background distribution (*p*-value > 0.05; Method). IS values were also compared between all pairwise bin combinations using two-sided Wilcoxon test. Significant differences were found only between bin 7 and bins 1 and 10 (for both comparisons *p-*value = 0.02 following the DFR correction). Thus, the decline in collaboration tendencies at the highest research overlap (right-most bin) can be explained, to a certain degree, by lower IS values. The substantial decline observed for the collaboration tendency with decreasing research overlap values—in comparison with the stability of the IS values along the same ROS range (bins 2–6 in Fig 1)—is constant and robust to the cutoffs and approaches used for calculating the research overlap score (S1 and S2 Figs). Hence, the drastic decline in collaboration tendency cannot be justified by the scientific quality derived from relatively distant interactions. This suggests that the preference to collaborate with researchers with similar scientific interests is not necessarily a rational one, but rather reflects gaps in communications between scientists from distant disciplines. Alternatively, growing variability in the scientific fields might introduce a gradient of limitations on the potential productive collaborations between researchers.

In addition to considering proximity in scientific interests, we next examined collaboration tendency as a function of potential social accessibility. To this end, we defined the following three non-overlapping social circles: researchers affiliated with the same department (intra-department), researchers affiliated with the same institute but not the same department (inter-department), and researchers affiliated with different institutions (inter-institutional). We then examined the tendency to collaborate (CLS score) and the quality of collaborations (IS score) in these circles. First, a similar pattern regarding the CLS-ROS interplay was observed across all social circles, showing a peak of collaboration at moderate-high ROS values (S3C Fig). Second, our data revealed that intra-departmental collaborations are significantly more frequent than inter-departmental and inter-institutional interactions (Table 1). This gradient of CLS scores, from higher to lower social accessibility, is supported by other studies which pointed out that researchers spend a substantial amount of research time with colleagues from their immediate work environment compared to persons outside it [32] and collaborate more with a decreasing physical distance [33]. By sorting scientists according to their relative seniority, determined according to their earliest last-name publication under their current affiliation within our data set, we further studied whether collaboration patterns changed with time. Though we note that caution should be taken when interpreting these results considering the limited time range of the screened publications and the simplistic strategy to determine seniority groups, we observed that inter-institutional collaborations are more frequent between the more senior scientists in comparison to their junior colleagues (S4 Fig). Finally, looking at the quality of the scientific work stemming from the above three social categories, the least rewarding interactions in terms of scientific impact occur between department members (Table 1). Hence, when considering both social accessibility and research overlap, the most practiced collaborations are not necessarily the most productive.

**Table 1:** Relative abundance and impact of collaborative interaction within different categories of affiliation associations.

	Inter- institutional (337,459 pairs) ^a^	Intra- institutional (30,819 pairs)	Within department (15,848 pairs)
**Collaboration relative abundance (number/fraction of collaborative interaction)**	216/6x10^-4^	158/0.005	669/0.04
**Mean Collaboration strength** ^b^ *	3.5x10^-5^	4x10^-4^	4x10^-3^
Mean ROS**	0.17	0.17	0.19
**Mean IS (number of interactions with recorded IS** ^**c**^ )***	6.3 (215)	5.1 (154)	4.6 (657)

^a^ Each pair is classified into a single category, e.g., the “within institutes” category does not include the “within department” group.

^b^ Mean collaboration strength was calculated as the average CLS for all pairs with at least a single co-authored article.

^c^ For some of the journals SJR score was not available. Mean IS was calculated only for interactions where (i) collaborative interaction was detected (CLS>0) and (ii) SJR score was available for at least a single journal where a joint publication appeared.

* Significant differences were observed between all categories (*p-* values in a Wilcoxon rank sum test < 10^−15^).

** Significant differences were observed between the “within department” category to the “intra- institutional” and “inter-institutional” categories (*p-*values in a Wilcoxon rank sum test < 10^−16^).

*** A significant difference was observed between all categories (*p-*values in a Wilcoxon rank sum test: “inter-institutional” versus “intra- institutional”– 5×10^−4^; “inter-institutional” versus “within departments”– 6×10^−12^; “within departments” versus “intra- institutional” –0.02).

## Conclusions

In previous studies, collaboration networks formed based on co-authorship in scientific articles were used to explore patterns, productivity and intensity of collaborations between researchers at different fields [e.g., 3,17,21], forming ‘scientific ecosystems’ and ‘scientific food webs’ [34]. Here, we systematically surveyed how personal gain can be optimized by balancing contradicting motivations accompanying the establishment of collaborative interactions. The repeatedly observed correlation [2,9,32] between the personal tendency of researchers to collaborate and the resulting scientific impact points at collaborative interactions as a beneficial professional pattern. Our results demonstrated that across different organization levels and disciplines within the biological sciences there is a remarkably low tendency to collaborate with other entities that are either too much or too little alike. The former can be related to a low synergistic potential, and in ecological sense suggests avoidance from competitive interactions; the latter can be related to a low likelihood of forming an interaction due to the lack of common ground. We note that by aggregating all co-authorship relations into a single dataset, we may have overlooked important differences across distinct collaboration types. In addition, our database was confined to researchers belonging to top research institutions, and might not be a representative sample of the academic community. As such, our results may not hold generally if a unique pattern of research interactions exist among the exclusive group of researchers we investigated.

Here, we observe that the reported CLS-ROS interplay is maintained in sub-sections of the data, stratified according to methodological discipline, model organism employed, or affiliation associations (S3 Fig). Beyond these classifications, the interplay between research overlap and collaboration tendencies may be different for regional versus international collaboration; in the former, frequent face-to-face meetings may more easily overcome scientific niche boundaries, while in the latter, larger overlap in scientific interests may be necessary to maintain productive collaboration. Similarly, different interactions are expected between mega projects (where each participant is expected to perform a familiar and well defined task without much interaction with other researchers) compared to small scale studies (where frequent flow of ideas is more beneficial). A richer dataset will be needed to examine such differences, as well as to compare the effect of gender, seniority, and specific research types (e.g., experimental versus theoretical) on scientific interactions. Similarly, the sampling of a wider time period (beyond the years 2000–2012 used for the current study) will allow exploring temporal effects of collaboration patterns including seniority, or occupational and field mobility.

Notably, competitive and cooperative interactions are not limited to the world of science and social interactions. In natural communities, species diversity is to a large extent being shaped by the interactions between co-occurring populations. An inverted U-shape relationship was suggested to describe the interplay between the cooperative potential and the degree of metabolic competition/resource overlap in microbial multi-species communities, where a moderate level of similarity in resource preferences of bacterial populations maximizes the potential for collaboration and higher or lower levels of resource overlap lead to a decline of the cooperative potential [35]. This relationship likely stems from the increasing competition on available resources, combined with the scarcity of differing resources that can be shared. The same principle also applies to economical models describing the likelihood of forming an inter-firm alliance versus the corresponding degree of technological overlap [36]. More generally, inverted U-shape relationships are typical of knowledge transfer networks relating the efficiency and productivity of industrial and academic initiatives with entities’ openness towards collaborations [37–39]. Our results, together with cross-discipline observations, point at the universality of the inverted U-shape principle for describing the tendency of collaborative interactions as a function of similarity of the niche (either physical or professional) pair members occupy. Whereas this pattern of alliances is intuitive, we further tested whether it is justified in light of actual scientific success. Our results indicated that the alliances that are formed between “unnatural” partners with regards to the inverted U-shape principle (i.e., those pairs with low research overlap) are typically not less successful than the “natural” ones. Moreover, we found that while accessibility encourages collaborations, productive scientific alliances tend to form when cooperative projects are established between scientists from different institutions. Overall, the current intuitive rational of researchers in the establishment of collaborative interactions is not necessarily the optimal strategy towards maximizing scientific quality.

## Additional Data Files

The following additional data are available with the online version of this paper. S1 Table lists the affiliations of researchers considered in the study; S1 and S2 Figs show the distribution of CLS and IS values across different level of pairwise research overlap (ROS), using alternative approaches for determining ROS; S3 Fig presents the fraction of collaborative interactions versus the level of research overlap in distinct scientific categories; S4 Fig presents the fraction of collaborative interactions formed by scientists of different seniority groups. Data assembled at this study were deposited at the Figshare repository (doi 10.6084/m9.figshare.1453090).

## Supporting Information

S1 Fig Distribution of pairwise scores for estimating the fraction of collaborative interactions (CLS) and their scientific impact scores (IS) across different level of pairwise research overlap (ROS), using multiple cutoffs for determining ROS. (PDF)

S2 Fig Repeating Fig 1 (main text) while using alternative approaches for calculating ROS. (PDF)

S3 Fig The fraction of collaborative interactions versus the level of research overlap in distinct scientific categories. (PDF)

S4 Fig The normalized fraction of collaborative interactions formed by scientists sorted by their seniority. (PDF)

S1 Table The number of researchers and their affiliations considered in this study. (PDF)
