# Supplementary material for: The Interplay between Scientific Overlap and Cooperation and the Resulting Gain in Co-Authorship Interactions
Source: PLoS One. 2015 Sep 15;10(9):e0137856. doi: 10.1371/journal.pone.0137856 (PMC4570763; doi:10.1371/journal.pone.0137856)

S1 Fig: Distribution of pairwise scores for estimating the fraction of collaborative interactions (CLS) and their scientific impact scores (IS) across different level of pairwise research overlap (ROS), using multiple cutoffs for determining ROS. The research overlap score between a pair of researchers is based on a list of MeSH terms constructed for each author. The list of MeSH terms was constructed by filtering those terms whose overall appearance in PUB_LAST is lower than a certain cutoff. The results presented in Figure 1 (main text) are based on setting this cutoff to 4. As shown here, other cutoffs yielded similar observations. The number at the top of each graph indicates the cutoff used (2,3,5 and 6). Dark grey (left bars) – Collaboration Score (CLS); Red (right bars) – Impact score (IS).

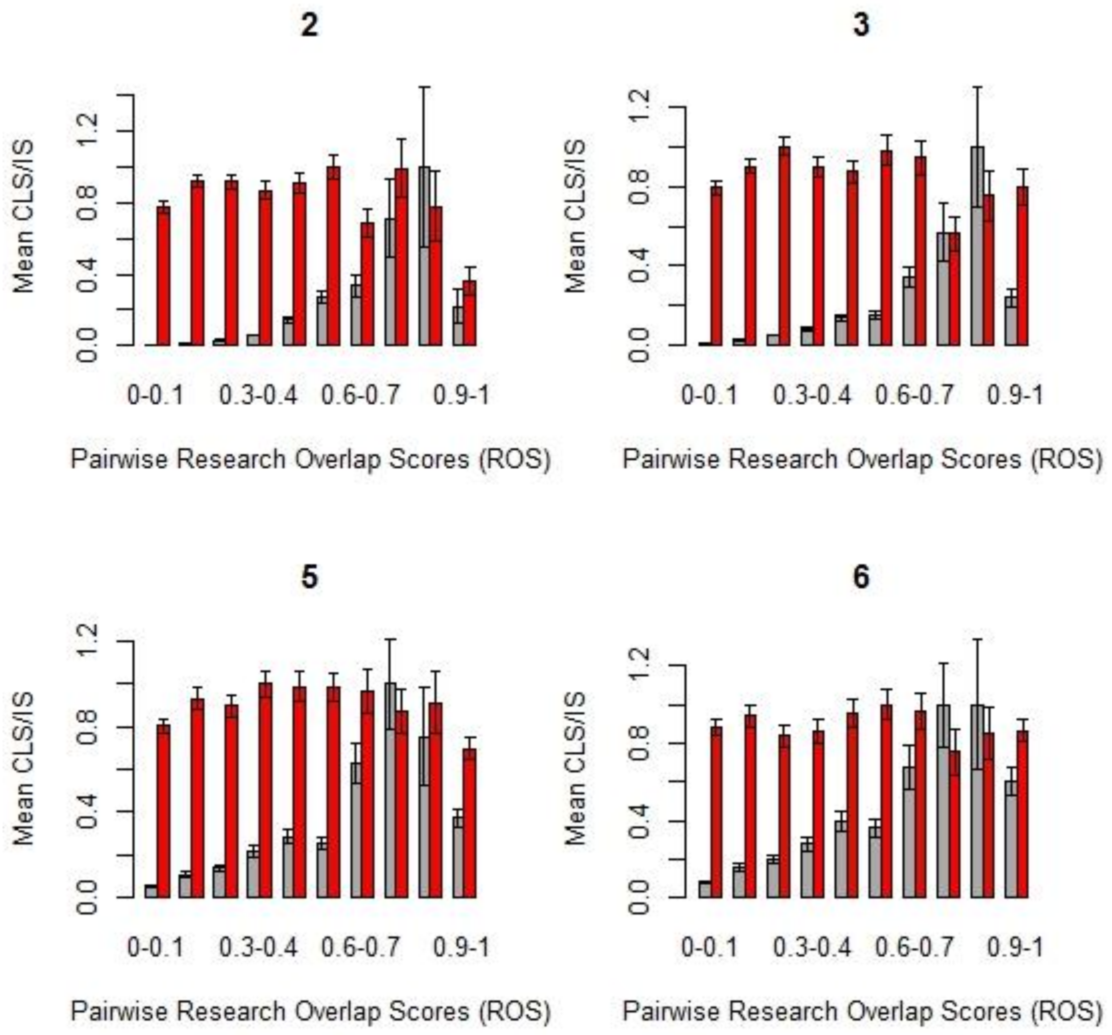

Supplement: S1 Fig — (PDF) [file pone.0137856.s001.pdf]
