# Supplementary material for: The Interplay between Scientific Overlap and Cooperation and the Resulting Gain in Co-Authorship Interactions
Source: PLoS One. 2015 Sep 15;10(9):e0137856. doi: 10.1371/journal.pone.0137856 (PMC4570763; doi:10.1371/journal.pone.0137856)
Supplement: S2 Fig — (PDF) [file pone.0137856.s002.pdf]

S2 Fig: Repeating Figure 1 (main text) while using alternative approaches for calculating ROS. For each alternative ROS measure, the distribution of collaborative interactions (CLS, dark grey bars) and the scientific impact scores (IS, red bars) is presented. CLS and IS are computed as in main text. ROS measures are computed using three alternative approaches to the one described in the main text. For all ROS measures, a cutoff of 4 appearances was set as the minimal threshold for associating a MeSH term with an author.

(A) For all possible pairwise combinations of authors, an a-symmetrical score was computed as follows:

$$ROS_{ij} = \sum_{n=1}^{N} \min\{f_{ni}, f_{nj}\}$$

where *N* is the set of all MeSH terms and f_{nx} is the fraction of MeSH term *n* across all papers associated with author *x*.

(B) The array of MeSH terms assigned for each author was restricted to the top ten most frequent MeSH terms (where each MeSH term has to appear more than a cutoff). An a-symmetrical research overlap score was computed as follows:

$$ROS_{ij} = \frac{\sum_{n \in P_i} 1(if\ n \in\ P_j)}{N_i}$$

where P_x is the full MeSH terms record in PUB_LAST of author *x* and N_x is the number of terms in that list. That is, the research overlap score of author *i* with author *j* is the fraction of their joint MeSH terms in PUB_LAST out of all terms selected to represent author *i*.

(C) The total variation distance, i.e., the normalized distance between two probability distributions. For comparison with other research overlap scores, we transformed the total variation distance into a similarity-based index by subtracting it from 1, so that a value of 1 presents maximal similarity and 0 maximal disparity.

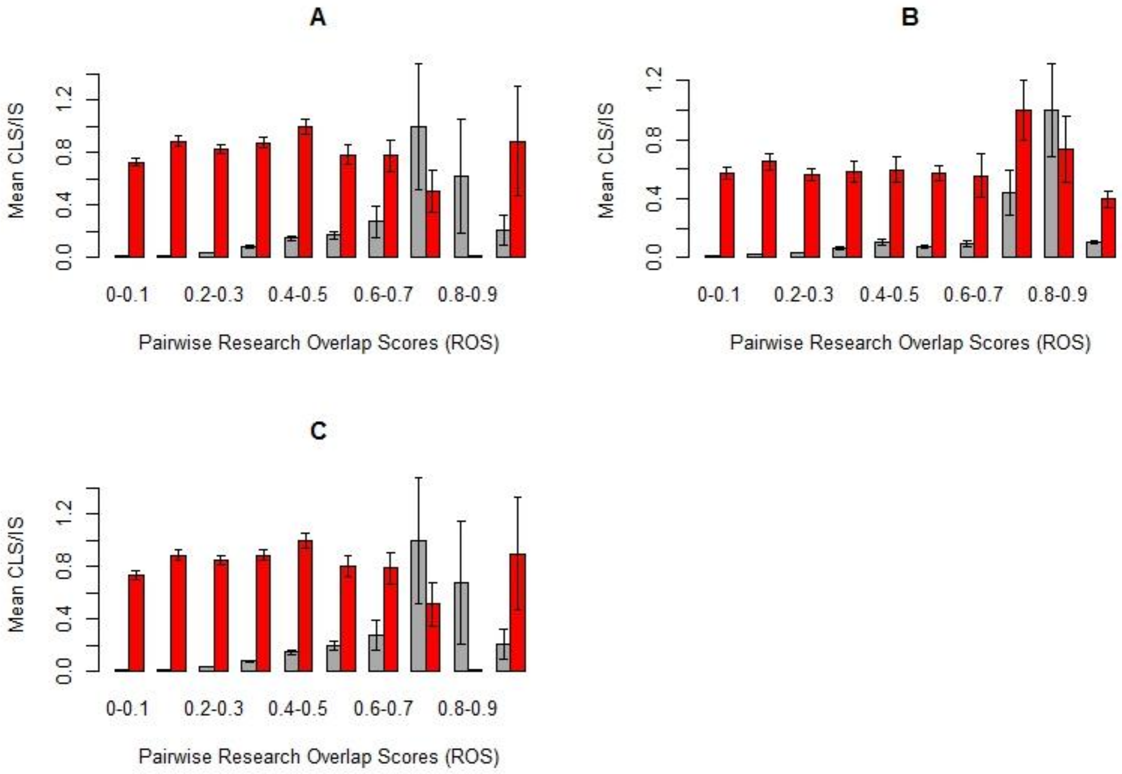
A
Mean CLS/IS
0.0
0.4
0.8
1.2
0-0.1
0.2-0.3
0.4-0.5
0.6-0.7
0.8-0.9
Pairwise Research Overlap Scores (ROS)
B
Mean CLS/IS
0.0
0.4
0.8
1.2
0-0.1
0.2-0.3
0.4-0.5
0.6-0.7
0.8-0.9
Pairwise Research Overlap Scores (ROS)
C
Mean CLS/IS
0.0
0.4
0.8
1.2
0-0.1
0.2-0.3
0.4-0.5
0.6-0.7
0.8-0.9
Pairwise Research Overlap Scores (ROS)
