# Supplementary material for: The Interplay between Scientific Overlap and Cooperation and the Resulting Gain in Co-Authorship Interactions
Source: PLoS One. 2015 Sep 15;10(9):e0137856. doi: 10.1371/journal.pone.0137856 (PMC4570763; doi:10.1371/journal.pone.0137856)

S3 Fig: The fraction of collaborative interactions versus the level of research overlap in distinct scientific categories. Distribution of pairwise scores for estimating the fraction of collaborative interactions (Collaboration Score, CLS) and their scientific impact scores (IS) across different levels of pairwise research overlap score (ROS). CLS values were normalized within each category by dividing the mean value for each ROS bin by the maximal mean CLS within each category. (A) Looking at the collaborations scientists tend to form when grouped according to methodological disciplines (Computational, Genetics, Molecular). Classification was carried by screening for the selected category in the associated MeSH terms of each researcher (considering MeSH terms with at least 4 appearances). Overall, 32, 125, and 191 scientists were assigned with MeSH terms containing the sub-strings ‘comput’, ‘genet’, and ‘molecular’, forming 28032, 109500, and 167316 non redundant pairwise combinations, respectively (all pairwise combinations of researchers in the group versus all researchers in our database). Number of pairs in bins (left to right): Computational – 21077, 4705, 1556, 483, 128, 49, 19, 8, 5, 2; Genetics- 69941, 22607, 9989, 4075, 1796, 746, 216, 81, 25, 24; Molecular- 111327, 30219, 14593, 5633, 2777, 1859, 436, 176, 29, 267 (B) Scientists were grouped according to selected model organisms (Bacteria, Plant, Human). Overall, 62, 45, and 393 scientists were assigned with MeSH terms containing the sub-strings ‘bacteria’ (or ‘microbial’), ‘plant’, and ‘human’, forming 54312, 39420, and 344268 non redundant pairwise combinations, respectively. Number of pairs in bins (left to right): Bacteria – 42158, 6786, 3480, 823, 345, 638, 17, 4, 15, 46; Plant – 33319, 2990, 2231, 517, 151, 124, 45, 11, 7, 25; Human – 169667, 76936, 43186, 23202, 10168, 8796, 3827, 1487, 884, 6115 (C) Scientists were grouped according their affiliation associations. Each pair is classified into a single category, e.g., the “Within university” category does not include the “Within department” group. The data includes 15848, 30819, and 337459 pairwise combinations of “Within department members”, “Within university”, and “Between Universities”, respectively. Number of pairs in bins (left to right): Between universities – 356315, 113349, 82029, 40770, 22674, 23985, 7725, 2501, 2002, 23568; Within university – 32237, 10552, 7718, 3645, 1925, 2260, 663, 261, 181, 2196; Within

department – 15303, 5560, 4199, 2022, 1350, 1142, 484, 194, 134, 1308. For clarity, bars (representing standard errors) were omitted from panels A-B due to the small sample size that resulted in large values.

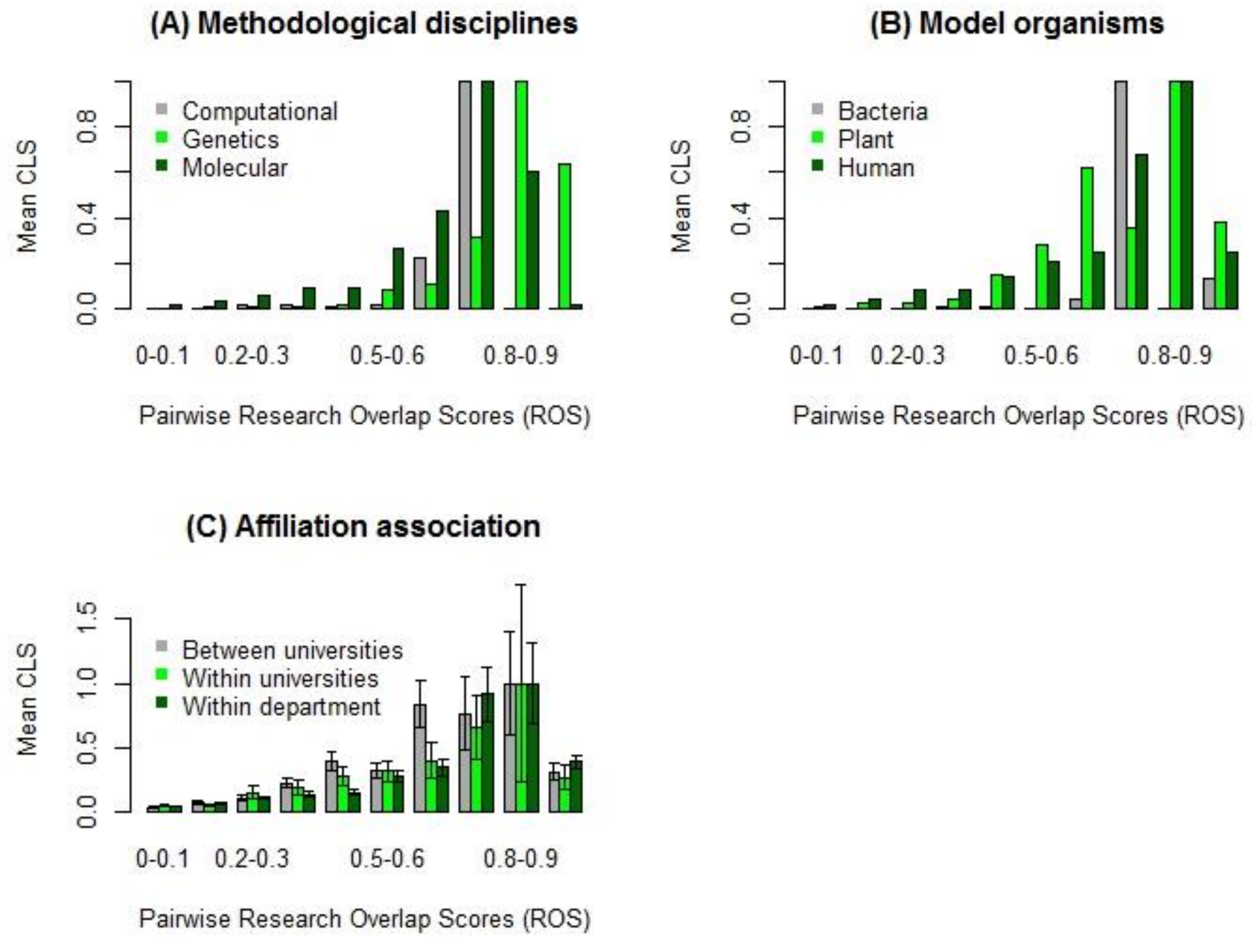

Supplement: S3 Fig — (PDF) [file pone.0137856.s003.pdf]
