# Supplementary material for: The Interplay between Scientific Overlap and Cooperation and the Resulting Gain in Co-Authorship Interactions
Source: PLoS One. 2015 Sep 15;10(9):e0137856. doi: 10.1371/journal.pone.0137856 (PMC4570763; doi:10.1371/journal.pone.0137856)
Supplement: S4 Fig — (PDF) [file pone.0137856.s004.pdf]

S4 Fig: The normalized fraction of collaborative interactions formed by scientists sorted by their seniority. The seniority of scientists was determined according to their earliest article in PUB_LAST. Senior scientists are these whose earliest article was published on 2006 or before; junior scientists are these whose earliest article was published between 2007-2012 (overall, 467 and 368 scientists, respectively). The non-uniform distribution of the earliest publication year prevented a more sparse distribution of the observations across the time axis. The mean CLS within each bar was normalized by division of the mean CLS value in the maximal mean CLS in the relevant group of affiliation associations. The normalization was done in order to conceal the large difference in the mean CLS between the different affiliation groups. Actual values are the following (left to right): 2.2×10^{-4}, 2.4×10^{-4} (all pairwise combinations); 3.8×10^{-5}, 3.3×10^{-5} (inter-institutional combinations); 4.6×10^{-3}, 6.0×10^{-3} (within department combinations). Statistical significance within the seniority groups in each group of affiliation-association was determined according to a two sided Wilcoxon test. Differences in the 'All combinations' and 'within department' are insignificant. Differences in the 'Inter-institutional' categories are significant ($p = 0.018$).

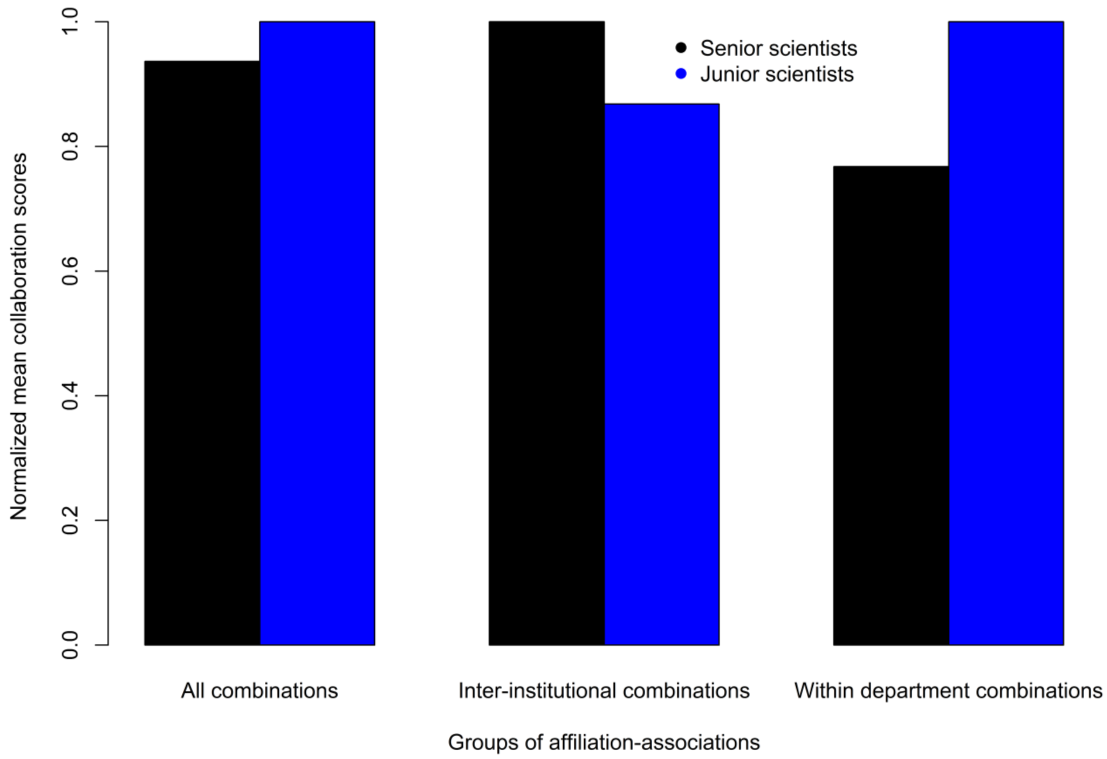
Senior scientists
Junior scientists
Normalized mean collaboration scores
0.0
0.2
0.4
0.6
0.8
1.0
All combinations
Inter-institutional combinations
Within department combinations
Groups of affiliation-associations
