# Supplementary material for: The Interplay between Scientific Overlap and Cooperation and the Resulting Gain in Co-Authorship Interactions
Source: PLoS One. 2015 Sep 15;10(9):e0137856. doi: 10.1371/journal.pone.0137856 (PMC4570763; doi:10.1371/journal.pone.0137856)
Supplement: S1 Table — (PDF) [file pone.0137856.s005.pdf]

S1 Table. The number of researchers and their affiliations considered in this study. A researcher can be affiliated with more than a single department.

University	Department	# of department members included in this study
BERKELEY	Integrative Biology	31
	Molecular and Cell Biology	76
CALTECH		40
CAMBRIDGE	Biochemistry	35
	Genetics	20
	Mitochondrial Biology Unit	6
	MRC Laboratory of Molecular Biology	51
	Physiology, Development and Neuroscience	31
	Plant Sciences	14
	Zoology	35
HARVARD	Human Evolutionary Biology	4
	Molecular and Cellular Biology	27
	Organismic and Evolutionary Biology	30
	Stem Cell and Regenerative Biology	9
MIT	Biology	86
	Biological Engineering	38
OXFORD	Plant Sciences	16
	Zoology	46
STANFORD	Chemical and Systems Biology	7
	Developmental Biology	15
	Genetics	31
	Microbiology and Immunology	24
	Molecular and Cellular Physiology	14
	Neurological Sciences	23
	Structural Biology	9
UCLA	Ecology and Evolutionary Biology	22
	Integrative Biology and Physiology	14
	Microbiology Immunology and Molecular Genetics	32
	Molecular Cell and Developmental Biology	13
UCSD	Biological Sciences	54
	Cell and Development Biology	18
	Ecology, Behavior and Evolution	7
	Molecular Biology	12
YALE	Cell Biology	20
	Cellular and Molecular Physiology	18
	Ecology and Evolutionary Biology	19
	Genetics	27
	Immunobiology	18
	Molecular Biophysics and Biochemistry	34
	Molecular Cellular and Developmental Biology	26
